# Proportion and Outcome of Induction of Labor Among Mothers Who Delivered in Teaching Hospital, Southwest Ethiopia

**DOI:** 10.3389/fpubh.2021.686682

**Published:** 2021-12-23

**Authors:** Tewodros Yosef, Dawit Getachew

**Affiliations:** Department of Epidemiology and Biostatistics, School of Public Health, College of Medicine and Health Sciences, Mizan-Tepi University, Mizan Teferi, Ethiopia

**Keywords:** bishop score, induction of labor, outcome, prevalence, Ethiopia

## Abstract

**Background:** Despite the induction of labor (IOL) having had some undesired consequences, it also has several benefits for maternal and perinatal outcomes. This study aimed to assess the proportion and outcome of IOL among mothers who delivered in Teaching Hospital, southwest Ethiopia.

**Methods:** A retrospective cross-sectional study was conducted from June 10 to June 20, 2019, among 294 mothers who gave birth between November 30, 2018, and May 30, 2019, by reviewing their cards using a structured checklist to assess the prevalence, outcome, and consequences of induction of labor. A binary logistic regression analysis was computed to look for the association between outcome variables and independent variables.

**Results:** The prevalence of labor induction was 20.4%. The most commonly reported cause of induction was preeclampsia (41.6%). The factors associated with IOL were mothers aged 25–34 years [AOR = 2.55, 95% CI (1.18–5.50)] and ≥35 years [AOR = 10.6, 95% CI (4.20–26.9)], having no history of antenatal care [AOR = 2.12, 95% CI (1.10–4.07)], and being Primipara AOR = 2.33, 95% CI (1.18–3.24)]. Of the 60 induced mothers, 23.3% had failed induction. The proportion of mothers with dead fetal outcomes and maternal complications was 5 and 41.7%, respectively. The unfavorable Bishop Score before induction [AOR = 1.85, 95% CI (1.32–4.87)] and induction using misoprostol [AOR = 1.48, 95% CI (1.24–5.23)] were the factors associated with failed induction of labor.

**Conclusion:** The prevalence of induced labor was considerably higher than rates in other Ethiopian studies; however, the prevalence of induction failure was comparable to other studies done in Ethiopia. The study found that Bishop's unfavorable score before induction and induction using misoprostol was the factor associated with unsuccessful induction. Therefore, the health professionals should confirm the favorability of the cervical status before the IOL to increase the success rate of induction of labor.

## Introduction

Induction of labor (IOL) is an iatrogenic stimulation or initiation of uterine contraction before the spontaneous onset of labor, with or without rupture of the membranes (1, 2). Globally, the prevalence of labor induction varies widely across countries. It is more commonly done in developed countries than in developing countries (1, 3, 4). IOL is done to achieve vaginal delivery before spontaneous labor starts, and it is recommended when the benefits of childbirth outweigh the risk of continuing the pregnancy (3–7). Its indication includes the mother's medical condition, pregnancy-related hypertension, post-term pregnancy, premature rupture of membrane (PROM), and intrauterine fetal death (IUFD) (4).

The induction of labor remains a controversial concept. The IOL influences the women's birth experience. It can be less efficient and is, in general, more painful than spontaneous labor (4). IOL involves medical interventions; increases hospital costs and should, therefore, be limited to medically indicated cases (8). Induction sometimes fails with potential risks of a higher rate of operative vaginal childbirth, cesarean birth, excessive uterine activity, abnormal fetal heart rate patterns, uterine rupture, maternal water intoxication, delivery of a preterm infant due to incorrect estimation of dates, and, possibly, cord prolapsed (4, 8–15).

But, most of the time, IOL is directly linked to reducing maternal mortality because it has a potential benefit in preventing maternal complications and improving pregnancy outcomes (3, 16, 17). IOL may reduce the emotional burden on the mother associated with carrying a dead fetus, the slight possibility of chorioamnionitis, and disseminated intravascular coagulation when a dead fetus is retained for more than 5 weeks in the 2nd or 3rd trimester (18). The risk of fetal death is well-known to increase during post-term pregnancies. Hence, the IOL after 41 completed weeks of pregnancy should be done to prevent fetal or neonatal death (11, 19, 20). Besides, when properly indicated, the procedure should also reduce the need for a cesarean section (21–23). It has been suggested that regions with high rates of induced labor tend to have lower rates of cesarean section (19).

Ethiopia is also one of the least developed African countries in which maternal and prenatal morbidity and mortality rates remain very high (24, 25). This impedes efforts to achieve the 3rd sustainable development goal, which is health and wellness and effective action to improve the quality of health care (26). Induction of labor can reduce specific clinical circumstances contributing to a greater or lesser degree of urgency, like chorioamnionitis, which has high-risk death for both the mother and the neonate (27). Even though some shreds of evidence are available in Ethiopia regarding IOL (5, 28–30), all were conducted in the northern and central parts of Ethiopia, which did not truly reflect the magnitude and factors associated with IOL in the southwest parts of Ethiopia. This study aimed to assess the proportion and outcome of IOL among mothers who delivered in Teaching Hospital, southwest Ethiopia.

## Methods

### Study Design, Area, and Period

A retrospective cross-sectional study was conducted among 294 mothers who gave birth in Mizan-Tepi University Teaching Hospital (MTUTH) between November 30, 2018, and May 30, 2019. MTUTH established in 1986 is one of the oldest hospitals in the federal democratic republic of Ethiopia, found in Bench Sheko Zone in the southern nation's nationalities and people's regional state at roughly 574 km southwest of Addis Ababa, the capital city of Ethiopia, and 849 km from the regional capital Hawassa. It is a teaching hospital with a total of 596 employees (208 nurses, 45 public health officers, 67 medical doctors, and 276 supporting staff). The study was conducted from June 10 to June 20, 2019.

### Source and Study Populations

All women who gave birth at MTUTH in the specified period were the source population. All selected women who gave birth within the specified period at MTUTH were the study population. The cards with appropriate indication and complete maternal data were included. The cards containing incomplete information were excluded.

### Sample Size Determination and Sampling Technique

The sample size was determined using a single proportion population formula based on the assumption of the prevalence of induced labor at Wolliso St. Luke Catholic hospital to be 22.4% (28) with a 95% CI, a margin of error of 5%, and adding 10% for non-response rate compensation. The total calculated sample size was 294. A systematic random sampling technique was used to select the potential candidate cards for review. There were 1,760 mothers who were delivered in MTUTH from November 30, 2018, to May 30, 2019. First, the total delivery recorded (1,760) was divided by the total sample size (294) to get the sampling interval, which was 6. The random start was selected between 1 and 6 randomly. Finally, every 6 cards were reviewed from a registration book until the required sample size was obtained.

### Study Variables and Measurements

The dependent variables were the IOL and failed induction. The independent variables were age, residence, gravidity, parity, and history antenatal care follow-up, gestational age at induction, Bishop Score before induction, weights of the baby, and methods of induction.

#### Successful Induction

If a woman gave birth vaginally with or without the aid of an instrument after labor induction (31).

#### Failed Induction

If a woman delivers by C/S due to failure to acquire either adequate uterine contraction (≥3 contractions and duration lasting ≥40 s in the 10-min period) or failed to show favorable cervical changes (reaches at least 4 cm in dilatation and fully effaced) despite being on oxytocin drip for at least 6–8 h) (31).

A favorable Bishop score was defined as a Bishop score ≥ 9 (29).

### Data Collection Method and Tools

The data were collected through a review of records from patient cards, labor ward logbooks, discharge logbooks, and operation room log books using a structured checklist from June 10 to June 20, 2019. The checklist was composed of some sociodemographic and obstetric history profiles, mode of the labor onset, indication for induction of labor, outcomes of labor induction, and maternal complications after the induction. The quality of the data was maintained by training the data collectors and utilizing a structured and tested checklist. Furthermore, regular checks on the completeness and consistency of the data were carried out on a daily basis.

### Data Processing and Analysis

The data were first checked manually for completeness, then coded, and entered into SPSS version 21 for analysis. Descriptive statistics were used to describe the study variables. A binary logistic regression analysis was used to look for the association between outcome variables and independent variables. Variables with a *p*-value of < 0.25 in the bivariate logistic regression were included in the multivariable logistic regression. Finally, variables in the multivariable logistic regression analysis with a *p* < 0.05 were considered as significantly associated with the outcome variables.

## Results

### Sociodemographic Characteristics

Of the 294 cards reviewed, 120 (40.8%) and 151 (51.4%) of the mothers were found in the age group of fewer than 25 years and rural residents, respectively. Of the 294 reviewed cards, 168 (57%) of the mothers were multigravida and 211 (71.7%) had ANC follow-up, of which 44.1% of them were completed the recommended ANC follow-up visit by the World Health Organization properly (a 4 ANC follow-up visit) (Table 1).

**Table 1:** Sociodemographic and obstetric history of mothers who delivered from November 30, 2018 to May 30, 2019, in MTUTH, southwest Ethiopia.

**Variables**	**Categories**	**Frequency**	**Percent**
Age of mothers (years)	15–24	120	40.8
	25–34	130	44.2
	>34	44	15
Residence	Urban	143	48.6
	Rural	151	51.4
Gravidity	Primigravida	126	42.9
	Multigravida	168	57.1
Parity	Primipara	158	53.7
	Multipara	136	46.3
ANC follow up	Yes	211	71.8
	No	83	28.2
Number of ANC follow up (*n* = 211)	One visit	52	24.7
	Two visit	33	15.6
	Three visit	33	15.6
	Four visit	93	44.1

### Mode of Labor Onset and Indication for IOL

Of the 294 mothers who gave birth, 60 (20.4%) were induced. Of the 60 induced women, 25 (41.7%) for preeclampsia, 16 (26.7%) for premature rupture of membranes (PROM), and 11 (18.3%) for post-term pregnancy were the reasons mentioned for induction of labor. As for the method of induction, 45 (75%) of the women had been intravenously injected with oxytocin (Table 2).

**Table 2:** Mode of the labor onset and indication for labor induction among mothers who delivered from November 30, 2018, to May 30, 2019, in MTUTH, southwest Ethiopia.

**Variables**	**Categories**	**Frequency**	**Percent**
Mode of labor onset	Spontaneous	234	79.6
	Induced	60	20.4
Reason for induction (*n* = 60)	Preeclampsia	25	41.7
	Premature rupture of membrane	16	26.7
	Post-term pregnancy	11	18.3
	IUFD	3	5
	CHF/Hypertension/Diabetes	3	5
	Oligiohydraminos	2	3.3
Gestational age at induction	<42 weeks	49	81.7
	≥42 weeks	11	18.3
Bishop score before induction	<9	18	30
	≥9	42	70
Methods of induction (*n* = 60)	Oxytocin	45	75
	Misoprostol	15	25

*IUFD, intrauterine fetal death; CHF, congestive heart failure*.

### Outcomes of Labor Induction

Of the 60 mothers induced labor, 10 (16.7%) of them had failed induction [cesarean section (CS) deliver]. Of the 60 induced women, 25 (41.7%) had maternal complications. The commonest maternal complication was postpartum hemorrhage accounted for 52%, followed by 44% external genitalia tear (Table 3).

**Table 3:** Outcomes of induction among mothers who delivered from November 30, 2018, to May 30, 2019, in MTUTH, southwest Ethiopia.

**Variables**	**Categories**	**Frequency**	**Percent**
Mode of delivery after induction (*n* = 60)	Spontaneous vaginal delivery	46	76.6
	Instrumental vaginal delivery	4	6.7
	Cesarean section	10	16.7
APGAR score (*n* = 60)	<7	10	16.7
	≥7	50	83.3
Birth weight of newborns (*n* = 60)	<2.5 kg	13	21.7
	2.5–3.9 kg	35	58.3
	≥4 kg	12	20
Admission to NICU (*n* = 57)	Yes	11	19.3
	No	46	80.7
Reason for admission (*n* = 11)	Fetal distress	6	54.5
	Preterm	5	45.5
Maternal complications (*n* = 25)	Postpartum hemorrhage (PPH)	13	52
	External genitalia tear	11	44
	Uterine rupture	1	4

*APGAR, appearance, pulse, grimace, activity and respiration; CPD, cephalo-pelvic disproportion; Kg, kilogram; NICU, neonatal intensive care unit*.

### Factors Associated With IOL and Failed Induction

After adjusting age, residence, and parity as confounding factors, women aged 25–34 years [AOR = 2.55, 95% CI (1.18–5.50)], ≥35 years [AOR = 10.6, 95% CI (4.20–26.9)], having no history of ANC follow-up during pregnancy [AOR = 2.12, 95% CI (1.10–4.07)], and being Primipara [AOR = 2.33, 95% CI (1.18–4.57)] were the factors associated with IOL (Table 4).

**Table 4:** Factors associated with induction of labor (IOL) among mothers who delivered from November 30, 2018, to May 30, 2019, in MTUTH, southwest Ethiopia.

**Variables**	**Categories**	**Mode of labor onse**		**COR (95% CI)**	**AOR (95% CI)**	***P***
		**Spontaneous**	**Induced**			
Age	15–24	106	14	1	1	
	25–34	106	24	1.71 (0.84–3.49)*	2.55 (1.18–5.50)	0.017
	≥35	22	22	7.57 (3.36–17.1)**	10.6 (4.20–26.9)	<0.001
Residence	Rural	115	35	1	1	
	Urban	119	25	0.69 (0.39–1.23)*	1.39 (0.71–2.73)	0.340
Antenatal care visit	Yes	177	34	1	1	
	No	57	26	2.38 (1.31–4.29)**	2.12 (1.10–4.07)	0.024
Parity	Primipara	119	39	1.80 (0.99–3.24)*	2.33 (1.18–4.57)	0.014
	Multipara	115	21	1	1	

*AOR, adjusted odds ratio; CI, confidence interval; COR, crude odds ratio.*

*p < 0.25, **p < 0.05.

After adjusting gestational age at induction, bishop score before induction and methods of induction as confounding factors, unfavorable bishop score (<9) before induction [AOR = 1.85, 95% CI (1.32–4.87)], and induction by misoprostol [AOR = 1.48, 95% CI (1.24–5.23)] were the factors significantly associated with failed IOL (Table 5).

**Table 5:** Factors associated with failed IOL among mothers who delivered from November 30, 2018, to May 30, 2019, in MTUTH, southwest Ethiopia.

**Variables**	**Categories**	**Induction outcom**		**COR (95% CI)**	**AOR (95% CI)**	***P***
		**Success**	**Failure**			
Gestational age at induction	<42 weeks	36	5	1	1	
	≥42 weeks	14	5	0.38 (0.12–2.52)*	0.42 (0.15–3.85)	0.432
Bishop score before induction	<9	13	4	1.90 (1.12–3.26)**	1.85 (1.32–4.87)	0.013
	≥9	37	6	1	1	
Weight of baby (kilogram)	<2.5	12	3	1	1	
	2.5–3.9	26	5	1.30 (0.38–5.56)*	1.15 (0.18–6.89)	0.752
	≥4	12	2	1.50 (1.05–4.45)*	1.32 (0.94–2.56)	0.214
Methods of induction	Oxytocin	30	7	1	1	
	Misoprostol	20	3	1.56 (0.84–2.69)*	1.48 (1.24–5.23)	0.039

*AOR, adjusted odds ratio; CI, confidence interval; COR, crude odds ratio.*

*p < 0.25, **p < 0.05.

## Discussion

Labor induction continues to be a debatable concept. Some researchers have found that IOL occurs + at an appropriate time associated with positive maternal and perinatal outcomes (6, 17, 20, 32). While other studies have also shown that induction may be associated with adverse and undesirable effects of maternal and perinatal outcomes (3, 8). This study aimed to assess the proportion and outcome of IOL among mothers who delivered in Teaching Hospital, southwest Ethiopia.

The proportion of induced labor was 20.4%, 95% CI (15.8–25%). The finding was in line with 22.4% in Wolliso St. Luke Catholic hospital, Ethiopia (28) and 16.47% in the Nigerian tertiary maternity unit (13). This finding was higher than 9% in two public hospitals of Mekelle town, Ethiopia (30), 10.9% in Lemlem Karl hospital of Miachew town in Ethiopia (5), 11.5% in Catholic Maternity Hospital, Nigeria (32), 11.4% in Latin America (8), and 9.72% in Nepal (7). But it was lower than 32.7% in France (33). The variation observed could be due to the difference in sociodemographic and educational factors. Even though not assessed in this study, the cultural and religious differences may create a great variation.

The prevalence of failed induction was 16.7%, 95% CI (7.2–26.2%). This finding was in line with 25.4% in four teaching hospitals of Addis Ababa, Ethiopia (29), 21.4% in Jimma specialized hospital, Ethiopia (2), and 24.1% in Catholic Maternity Hospital, Nigeria (32). But it was lower than 29.5% in two public hospitals of Mekelle town, Ethiopia (30), 42.1% in Wolliso St. Luke Catholic hospital, Ethiopia (28), and 43.6% in Mahatma Gandhi Medical College and Research Institute Hospital, India (34). The variation observed could be due to the methods used during the induction of labor.

Pregnant women aged 25–34 and 35 years and above were 2.6 and 10.6 times more likely to be induced than those under 24 years, respectively. The increased age of pregnant women was statistically associated with the induction of labor. This finding was supported by studies done elsewhere (8, 35, 36). This could be due to the need to interrupt the pregnancy associated with intrauterine fetal death in women of older age (37, 38).

Pregnant women who did not have an ANC follow-up visit were two times as likely to undergo induction as those who had an ANC follow-up visit. This finding was supported by Rade et al. (5) and Abdulkadir et al. (28). Sandall et al. also said that women with midwife-led pregnancies are more likely to give birth without being induced but have natural labors (39). This could be because of the higher proportion of mothers who diagnosed with post-term pregnancy and intrauterine fetal deaths are associated with no history of ANC visit. That may increase the chance of induced labor among non-ANC followers.

Being primigravida has been significantly linked to labor induction. Primigravida women were 2.3 times more likely to have labor induction than multigravida women. This finding was consistent with a study done by O'Dwyer et al. which revealed that primigravida was more likely to have labor induced than multigravida (40). Another study revealed that most of the induction was done in the younger age group with primigravida (7).

A pregnant woman with an unfavorable Bishop score before induction had 1.9 times increased odds of having failed induction than those with a favorable bishop score before induction. In this study, an unfavorable Bishop score (<8) was very strongly associated with the failed induction of labor. This finding was supported by studies conducted in Ethiopia (2, 28–30). Similarly, a study was done by Devarasetty et al. revealed that vaginal delivery after induction was high in pregnant women with a Bishop score ≥5 before induction (34). A high Bishop score means that there is a greater chance that an induction will be successful. If the score is 8 or above, it is a good indication that spontaneous labor would start soon (41).

Pregnant women who are induced by misoprostol were 1.5 times more likely to have failed induction than those who are induced by oxytocin. The use of oxytocin was strongly associated with a higher success rate of induction of labor. This study was supported by Vogel et al. that revealed that induction by oxytocin alone is associated with over an 80% success rate (3). A study done by Scapin SQ et al. revealed that induction by misoprostol was more associated with vaginal delivery (42). But another study concludes that the success rate of vaginal delivery after induction was very similar for oxytocin and misoprostol (8). The discrepancy finding was revealed in the observational study designs. To clear these two opposing ideas, a randomized control trial should be considered.

## Limitations of The Study

The main limitation of this study could be a study based on secondary data where the quality of documentation, data, and record keeping are questionable. The exclusion of cards with incomplete data may introduce a selection bias.

## Conclusion

The prevalence of induced labor was considerably higher than rates in other Ethiopian studies; however, the prevalence of induction failure was comparable to other studies done in Ethiopia. The study found that Bishop's unfavorable score before induction and induction using misoprostol was the factor associated with unsuccessful induction. Therefore, the health professionals should confirm the favorability of the cervical status before the IOL to increase the success rate of induction of labor.

## Data Availability Statement

The original contributions presented in the study are included in the article/supplementary material, further inquiries can be directed to the corresponding authors.

## Ethics Statement

The studies involving human participants were reviewed and approved by Mizan-Tepi University Institutional Review Board. The patients/participants provided their written informed consent to participate in this study.

## Author Contributions

TY and DG involved in the conception, design, and acquisition of data, analysis, and interpretation of the results. TY drafted the manuscript and approved it for publication. All authors contributed to the article and approved the submitted version.

## Conflict of Interest

The authors declare that the research was conducted in the absence of any commercial or financial relationships that could be construed as a potential conflict of interest.

## Publisher's Note

All claims expressed in this article are solely those of the authors and do not necessarily represent those of their affiliated organizations, or those of the publisher, the editors and the reviewers. Any product that may be evaluated in this article, or claim that may be made by its manufacturer, is not guaranteed or endorsed by the publisher.

## Abbreviations

ANC: antenatal care
AOR: adjusted odds ratio
CI: confidence interval
COR: crude odds ratio
IOL: induction of labor
MTUTH: Mizan Tepi University Teaching Hospital
SPSS: Statistical Package for the Social Sciences.
